# Current Concepts and Future Applications of Non-Invasive Functional and Anatomical Evaluation of Coronary Artery Disease

**DOI:** 10.3390/life12111803

**Published:** 2022-11-07

**Authors:** Evangelos Oikonomou, Panagiotis Theofilis, Stamatios Lampsas, Ourania Katsarou, Konstantinos Kalogeras, Georgios Marinos, Aikaterini Tsatsaragkou, Artemis Anastasiou, Antonios Lysandrou, Maria-Ioanna Gounaridi, Ioannis Gialamas, Michael-Andrew Vavuranakis, Dimitris Tousoulis, Manolis Vavuranakis, Gerasimos Siasos

**Affiliations:** 1 3rd Department of Cardiology, Medical School, National and Kapodistrian University of Athens, Sotiria Chest Disease Hospital, 11527 Athens, Greece; 2 1st Department of Cardiology, Medical School, National and Kapodistrian University of Athens, Hippokration General Hospital, 11527 Athens, Greece; 3 Department of Hygiene, Epidemiology and Medical Statistics, School of Medicine, National and Kapodistrian University of Athens, 11527 Athens, Greece; 4 Cardiovascular Division, Harvard Medical School, Brigham and Women’s Hospital, Boston, MA 02115, USA

**Keywords:** coronary artery disease, non-invasive, anatomical, functional, atherosclerotic lesion

## Abstract

Over the last decades, significant advances have been achieved in the treatment of coronary artery disease (CAD). Proper non-invasive diagnosis and appropriate management based on functional information and the extension of ischemia or viability remain the cornerstone in the fight against adverse CAD events. Stress echocardiography and single photon emission computed tomography are often used for the evaluation of ischemia. Advancements in non-invasive imaging modalities such as computed tomography (CT) coronary angiography and cardiac magnetic resonance imaging (MRI) have not only allowed non-invasive imaging of coronary artery lumen but also provide additional functional information. Other characteristics regarding the plaque morphology can be further evaluated with the latest modalities achieving a morpho-functional evaluation of CAD. Advances in the utilization of positron emission tomography (PET), as well as software advancements especially regarding cardiac CT, may provide additional prognostic information to a more evidence-based treatment decision. Since the armamentarium on non-invasive imaging modalities has evolved, the knowledge of the capabilities and limitations of each imaging modality should be evaluated in a case-by-case basis to achieve the best diagnosis and treatment decision. In this review article, we present the most recent advances in the noninvasive anatomical and functional evaluation of CAD.

## 1. Introduction

Coronary artery disease (CAD) is one of the major cardiovascular diseases (CVD) and one of the major causes of death. The pathophysiologic background of CAD is atherosclerosis which is considered a chronic process of vascular pathology. Atherosclerotic lesions are progressively evolved in the vascular wall of small, medium and large arteries by the formation of lipid-rich plaques and a state of perpetuating inflammation which are thought to begin in early adulthood [1]. In the early days of invasive coronary angiography; the precise quantification of luminal narrowing was challenging, which is not considered a barrier today [2]. However invasive coronary angiography hardly gives information on the condition of the vessel wall or on atherosclerotic plaque futures and structures [3].

Invasive coronary angiography remains the gold standard for epicardial coronary arteries luminal imaging, and for diagnosis, and evaluation of the degree of luminal stenosis [4]. However, not only lumen stenosis but also plaque volume and plaque characteristics serve a critical role in the prognosis of patients with CAD [3]. Non-invasive functional imaging has significantly developed over the last decades in the fields of dobutamine stress echocardiography (DSE), and single photon emission computed tomography (SPECT). Additional functional and anatomical imaging modalities, i.e., computed tomography (CT) coronography, CT myocardial perfusion, CT instantaneous wave-free ratio (CT-iFR) and fractional flow reserve (CT-FFR) and cardiac magnetic resonance (MRI) stress imaging and perfusion, have been introduced in the clinical armamentarium in the last decade.

Therefore, in this review article, we discuss the role of different functional and anatomical non-invasive imaging techniques to establish the diagnosis, to assess the prognosis and to guide the treatment of patients with coronary artery disease.

## 2. Prognostic Value and Treatment of Anatomical and Functional Significant CAD Lesions

### 2.1. Progression to Vulnerable Coronary Atherosclerotic Lesion

Coronary artery atherosclerosis is a complex process, which leads to the development of atherosclerotic plaques that may cause clinically significant coronary events by rupture, erosion, hemorrhage or by a progressive increase to clinically significant lumen stenosis [5]. Endothelial dysfunction causes low-density lipoprotein (LDL) molecule internalization, resulting in subintimal deposition of lipids [6]. Endothelial cell (EC) dysfunction serves a vital role in the various manifestations induced by the atherosclerotic process as a vast, selectively permeable interface, regulating the transport of fluid and macromolecules via an elaborate system of transcellular vesicles and intercellular junctional complexes [7]. Accumulation of LDLs in the intima act as the chronic stimulator of the innate and adaptive immune response, triggering vascular smooth muscle cells (VSMCs) and ECs to express adhesion molecules, growth factors and chemo-attractants that facilitate leukocytes to adhere to arterial endothelium, to penetrate the endothelial cells and to scavenge lipid particles, becoming foam cells [8]. Foam cells serve as a reservoir of esterified cholesterol in lipid droplets, promoting the further release of growth factors and cytokines and VSMC migration from the media into the intima where they contribute to the release of lipids into the extracellular space, forming acellular “lipid pools” [9]. At the same time, activated VSMCs in the intima proliferate and increase their collagen production by making thick fibrotic layers of connective tissue, the “fibrous cap” [10]. After the establishment of the atherosclerotic plaque, its deeper layers may become hypoxic triggering angiogenesis and microvessel proliferation of the “vasa vasorum” [11].

**Figure 1:**
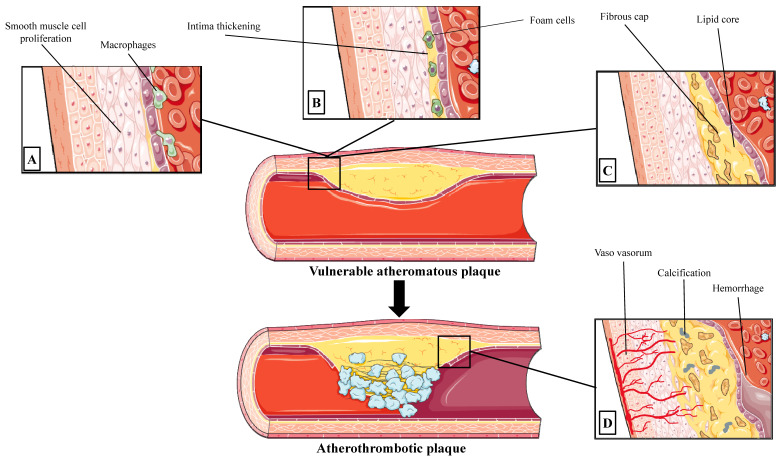
Characteristics of vulnerable atheromatous plaque and atherothrombotic plaque. Panel (**A**) Dysfunction of the endothelial cells and accumulation of low-density lipoproteins in the intima affects the permeability of endothelium, resulting in macrophage infiltration and vascular smooth muscle cells (VSMCs) proliferation; Panel (**B**) Macrophages become foam cells promoting further VSMCs proliferation and enlargement of “lipid core”; Panel (**C**) Neoangiogenesis and microvessel proliferation of the “vasa vasorum” is detected in the vulnerable plaques. Moreover, thin fibroatheromas are the likely precursors of fatal coronary plaque ruptures. Panel (**D**) Calcific lesions and intraplaque hemorrhage can lead to lesion destabilization, which can lead to consequent coronary thrombotic events. “Parts of the figure were drawn by using pictures from Servier Medical Art. Servier Medical Art by Servier is licensed under a Creative Commons Attribution 3.0 Unported License (https://creativecommons.org/licenses/by/3.0/, accessed on 10 September 2022)”.

However, atherosclerotic plaques during this complex process of atherogenesis can be destabilized during several of these atherogenetic steps. Plaques’ vulnerability is common with a decrease in the smooth muscle cells and extracellular matrix content, as in the cases of the large necrotic core with a thin fibrous cap [12]. Thin-cap fibroatheromas are the likely precursors of up to 83% of fatal coronary plaque ruptures [12]. Mechanical weakening of the fibrous cap is an important step that precedes plaque rupture and consequent secondary thrombotic events [13]. The restoration of lesional cell death and apoptotic cells is a complex process which is conducted by phagocytes that serves a vital anti-inflammatory role, a procedure that is termed efferocytosis [14]. Dysregulation of the efferocytosis reduces apoptotic cell clearance, resulting in plaque enlargement, further necrotic core formation and plaque rupture [15]. Moreover, hypoxia on advanced atherosclerotic lesions induces neoangiogenesis and positive remodeling, a signature process crucially contributing to the weakening of the plaque [16]. Vasa vasorum of thin-walled coronary atherosclerotic arteries, contributes to the structural integrity of the microvascular endothelium and are another point of concern due to its ability to cause intraplaque hemorrhage and secondary thrombotic events [16]. Therefore, several molecular and mechanical processes can lead to lesion destabilization and plaque vulnerability, and an attempt for classification was conducted by the American Heart Association classification (types I–VI) that serves a sequence of histological lesion progression (Figure 1).

### 2.2. Types of Atherosclerotic Lesion in Acute Coronary Syndromes

For many years, it is highly believed that most acute coronary syndromes are due to the rupture of mildly stenotic plaques, which is based on invasive angiographic studies which evaluate the lumen’s stenosis in patients whose coronary artery disease progresses to myocardial infarction [17]. Although, postmortem findings of patients who died suddenly due to acute coronary syndrome revealed that infarct-related lesions in acute myocardial infarction exhibited more than a 75% degree of coronary area narrowing [18]. While plaque rupture is one of the major mechanisms of coronary acute thrombotic events, plaque erosion is recognized with increasing frequency [19]. Contemporary data support that plaques with thin fibrous caps and large lipid pools frequently rupture and cause acute clinical events. However, lesions rich in extracellular matrix detected by optical coherence tomography (OCT), which is characterized as plaque erosion, accounted for up to 31% of all coronary clinical cases [20]. In that way, this large study revealed that, in patients with acute coronary events, 64% of the patients displayed plaque rupture, 27% of them plaque erosion and 8% demonstrated calcified nodules [20].

### 2.3. Atherosclerotic Lesions in Stable CAD

Significant CAD is detected by atherosclerotic plaque accumulation in the epicardial arteries, whether obstructive or non-obstructive [4]. CAD is a dynamic process of atherosclerotic plaque accumulation and functional alterations of coronary circulation that can be modified by lifestyle, pharmacological therapies and revascularization, which result in disease stabilization or regression [4]. Chronic CAD syndromes are characterized by stabilized atheromas with thickened fibrous caps and an increasing proportion of densely calcified plaque [21]. In contrast to acute coronary syndromes that are commonly developed in mild lumen stenosis, in chronic CAD, patients have hemodynamically significant stenosis (FFR, ≤0.80). Both the FAME 2 trial and the PROSPECT (Providing Regional Observations to Study Predictors of Events in the Coronary Tree) study showed that the main determinants of future events in stable lesions were a small luminal area, and that angioplasty in sites of severe lesions may not prevent future myocardial infarction [22,23].

### 2.4. Functional Directed Treatment of CAD

In acute coronary syndromes (ACS) the value and prognostic significance of emergent revascularization have been proven in most of the cases [24,25]. However, this is not always the case in chronic coronary syndromes [4]. Indeed, revascularization in patients with chronic coronary syndromes is superior to medical management alone, especially in the relief of symptoms but not always regarding the patient’s prognosis [4]. Identification and functional assessment of ischemia can provide prognostic information. Indeed, abnormal nuclear perfusion scans, especially when ischemia extends to at least 10% of the myocardium confer an adverse prognosis [26,27]. Similar to nuclear perfusion other imaging modalities can provide an assessment of myocardial perfusion and when significant ischemia is detected with stress echocardiography portents an adverse prognosis [28].

However, as it was shown in the ORBITA trial in patients with stable angina and single vessel disease with luminal diameter stenosis >70% revascularization did not improve symptoms over medical treatment [29]. The recently published ISCHEMIA trial in patients with moderate or severe ischemia, as it was assessed by nuclear perfusion imaging, stress echocardiography, stress cardiac magnetic resonance imaging and exercise stress test, does not reveal any difference between initial conservative management over initial revascularization strategy regarding the cardiovascular (CV) events risk or death [30].

Of course, the most direct method to identify coronary luminal diameter stenosis is a visual assessment by coronary angiography. However, the DEFER trial documented that percutaneous coronary intervention (PCI) has no benefit over conservative management in patients with intermediate coronary stenoses if fractional flow reserve (FFR) was estimated ≥0.75 [31,32]. Nevertheless, in patients with chronic coronary syndrome and multivessel coronary artery disease revascularization if FFR ≤ 0.80 improve patients’ outcomes and prognosis [33]. Moreover, the correlation between coronography assessment of stenoses and hemodynamic significance was poor (35%) in lesions estimated to cause 50–70% stenoses while 20% of stenoses estimated 71–90% were not hemodynamically significant. The FAME2 trial expands the knowledge on the importance of functional assessment to the decision of treatment strategy. Indeed, in patients with chronic coronary syndromes, and at least one lesion with FFR ≤ 0.80, treatment with percutaneous coronary intervention and best medical treatment over best medical treatment alone improve the patients’ outcomes [34].

Since treatment decisions should be based on the best available data as early as possible in the course of the disease, and with the best and less invasive approach, the noninvasive functional and anatomical evaluation of coronary artery disease must be elaborated in clinical practice (Table 1).

## 3. The Role of Biomarkers in the Assessment of CAD

Atherosclerotic lesions in the CAD are dynamic, as they grow over time, cause ischemia and, finally, cause obstructive events [35]. Circulating biomarkers have a variety of clinical applications on CAD diagnosis in multiple stages of the disease [36,37,38]. Acute CAD events are correlated with the measurement of myocardial necrosis biomarkers, such as Creatine-Kinase-MB isoform and high-sensitivity Cardiac Troponin, which are released when irreversible myocardial damage occurs [39]. Moreover, natriuretic peptides such as N-terminal pro-B-type natriuretic peptide (NT-proBNP) which is an established biomarker for prognostication and treatment of CAD are clinically useful [40]. Inflammatory biomarkers are also used for diagnosis and prognosis and are found elevated in unstable CAD [41,42]. C-reactive protein, Interleukin-6 and adhesion molecules, such as Intercellular Adhesion Molecule 1 (ICAM-1), Vascular Cell Adhesion Molecule 1 (VCAM-1) and Selectins, have been observed to be related to adverse cardiovascular prognosis [43,44,45]. Recently, the role of small (18–22 nucleotides) non-coding microRNAs has been studied in several disease states and pathophysiologic conditions and several patterns of circulating or tissue microRNAS have been recognized in patients with extensive atherosclerosis, CAD and acute coronary syndromes [46,47,48,49,50]. Similarly, the role of cell-free highly fragmented double-stranded DNA, circulating in the serum, is found in high levels in patients with myocardial infarction. Moreover, there is a significant interrelationship between cardiac troponin levels and cell-free DNA and when these two biomarkers are combined may give significant insights into the progress to heart failure following an acute coronary syndrome [51].

## 4. Computed Tomography

**Figure 2:**
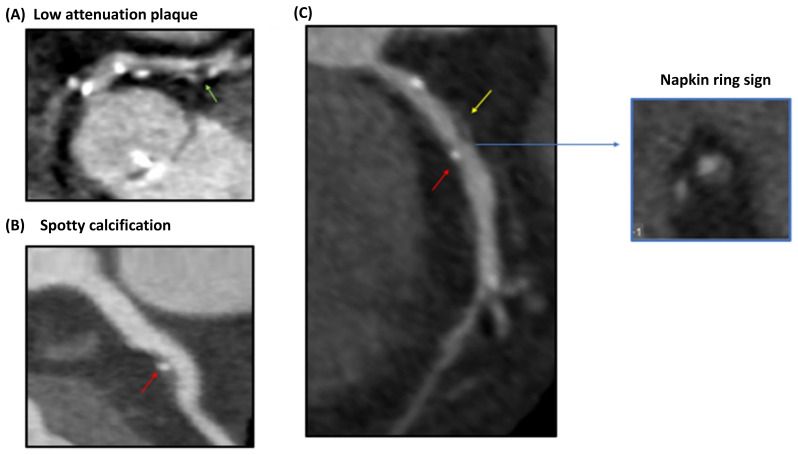
Coronary CT angiography findings of high-risk coronary atherosclerotic plaques. Certain identifiable patterns are indicative of a vulnerable plaque phenotype, namely (**A**) low plaque attenuation, (**B**) spotty calcification, and (**C**) the napkin ring sign. Reproduced with permission from Daghem, M. et al. [59], British Journal of Pharmacology; published by John Wiley & Sons Ltd., 2021, used under Creative Commons CC BY 4.0 license. HU: Hounsfield units. Yellow arrow indicates positive remodeling, red arrow indicates spotty calcification, blue arrow indicates the napkin ring sign.

Coronary CT angiography (CCTA) has become popular as a non-invasive method of assessing CAD diagnosis and prognosis (Figure 2). Early reports pointed to the usefulness of the 16-slice CCTA, which provided accurate assessments in 88.4% of the coronary artery segments [52]. The sensitivity of the technique was lower when evaluating distal and secondary branches of major coronary arteries. For patients with an intermediate pre-test probability for CAD, CCTA is a reliable option for ruling out CAD (negative predictive value 99%), with 64-slice CCTA being slightly superior compared to 16-slice CCTA [53]. Similar accuracy regarding the detection of ≥50% or ≥70% stenosis was reported in the Assessment by Coronary Computed Tomographic Angiography of Individuals Undergoing Invasive Coronary Angiography (ACCURACY) prospective trial [54]. According to a contemporary study, the use of CCTA could safely reduce the need for invasive coronary angiography from 100 to 14%, with identical rates of long-term adverse CV events between patients randomized by CCTA and invasive coronary angiography [55]. CCTA’s diagnostic accuracy for obstructive CAD may approach invasive coronary angiography using contemporary 320-slice scanners (per-patient analysis area under receiver operating characteristics curve (AUROC): 0.90, per-vessel analysis AUROC: 0.87, per-segment analysis AUROC: 0.81) [56]. Most importantly, individuals with stable chest pain and intermediate pre-test probability randomized to CCTA exhibited similar rates of major adverse CV events after 3.5 years of follow-up compared to those submitted to invasive coronary angiography [57]. Recent advances in artificial intelligence may further aid CCTA, with the study of the CREDENCE substudy analysis pointing to a high agreement between the artificial intelligence-based evaluation of CCTA and invasive coronary angiography [58]. Other than the evaluation of anatomical stenosis, cardiac CT and CCTA may provide additional information that will be discussed below.

Coronary CT angiography (CCTA) has become popular as a non-invasive method of assessing CAD diagnosis and prognosis (Figure 2). Early reports pointed to the usefulness of 16-slice CCTA, which provided accurate assessments in 88.4% of the coronary artery segments [52]. The sensitivity of the technique was lower when evaluating distal and secondary branches of major coronary arteries. For patients with an intermediate pre-test probability for CAD, CCTA is a reliable option for ruling out CAD (negative predictive value 99%), with 64-slice CCTA being slightly superior compared to 16-slice CCTA [53]. Similar accuracy regarding the detection of ≥50% or ≥70% stenosis was reported in the Assessment by Coronary Computed Tomographic Angiography of Individuals Undergoing Invasive Coronary Angiography (ACCURACY) prospective trial [54]. According to a contemporary study, the use of CCTA could safely reduce the need for invasive coronary angiography from 100 to 14%, with identical rates of long-term adverse CV events between patients randomized to CCTA and invasive coronary angiography [55]. CCTA’s diagnostic accuracy for obstructive CAD may approach invasive coronary angiography using contemporary 320-slice scanners (per-patient analysis area under receiver operating characteristics curve (AUROC): 0.90, per-vessel analysis AUROC: 0.87, per-segment analysis AUROC: 0.81) [56]. Most importantly, individuals with stable chest pain and intermediate pre-test probability randomized to CCTA exhibited similar rates of major adverse CV events after 3.5 years of follow-up compared to those submitted to invasive coronary angiography [57]. Recent advances in artificial intelligence may further aid CCTA, with the study of the CREDENCE substudy analysis pointing to a high agreement between the artificial intelligence-based evaluation of CCTA and invasive coronary angiography [58]. Other than the evaluation of anatomical stenosis, cardiac CT and CCTA may provide additional information that will be discussed below.

### 4.1. Coronary Artery Calcium

Among the most frequent investigations in the field of cardiac CT is the measurement of the coronary artery calcium (CAC) score, a surrogate marker of coronary atherosclerosis burden. According to the multi-ethnic study of atherosclerosis of 6814 participants free of CVD, a CAC score of 0 was associated with 10-year event rates below 5%. In cases of a CAC score above 300, the event rates ranged from 13.1 to 25.6% [60]. Interestingly, individuals with very high low-density lipoprotein cholesterol (≥190 mg/dL) and a zero CAC score may have a low risk of incident CV events, despite being considered a high-risk group [61]. CAC score progression does not provide any incremental prognostic value based on the findings of Lehmann et al., in 3821 individuals free of CVD [62]. Such findings signify the importance of zero CAC score as a negative risk factor for incident adverse CV events. Moreover, in a recently published study, the addition of CAC on top of available risk scores such as the Multi-Ethnic Study of Atherosclerosis risk score and the Pooled Cohort Equations risk score provided superior discriminating capacity concerning CAD and CVD prediction, especially in subjects with a borderline risk [63]. It should be stressed, however, that the exclusion of patients with a CAC score of 0 could lead to no diagnosis of microvascular dysfunction in a considerable proportion of patients, who may thus face a high risk of mortality [64].

### 4.2. Vulnerable Plaque Assessment

Apart from the evaluation of the anatomical stenosis, the identification of vulnerable plaque characteristics is also of importance. Multi-slice computed tomography (MSCT) has been investigated extensively in this regard, particularly in the detection of thin cap fibroatheromas (TCFAs). A mixed plaque morphology in MSCT may signify the presence of a TCFA [65]. Positive remodeling and low plaque attenuation values are additionally important characteristics as they have been associated with OCT-detected TCFAs in culprit lesions [66]. Moreover, TCFAs may present with a ring-like enhancement, with this finding possessing a limited diagnostic accuracy, however [66]. In patients with clinically suspected CAD who underwent OCT and MSCT, an attenuation value of ≤62.4 Hounsfield Units (HU), a remodeling index ≥ 1.08 and a signet ring-like enhancement on MSCT were predictive of OCT-defined TCFA after adjustment for confounders [67]. Among those features, plaque attenuation values may yield the greatest diagnostic potency (AUROC 0.859) [67]. Last but not least, an increase in epicardial fat volume and density could also be a sign of TCFA presence [68,69]. When differentiating between patients with acute coronary syndrome (ACS) or stable CAD, a non-calcified or mixed plaque was frequently observed in patients with ACS in contrast to the calcified plaques seen in those with stable CAD [65]. Other high-risk features, namely positive remodeling and spotty calcification may be associated with ACS compared to stable CAD [70].

### 4.3. Perivascular Fat Attenuation Index

The evaluation of coronary perivascular adipose tissue (PVAT), through imaging techniques, has been recently proposed, with the so-called perivascular fat attenuation index (FAI) through a CCTA. Through this method, one can assess the adipocyte lipid content and size, which is believed to indicate the degree of vascular inflammation, similar to cardiac PET [71]. Perivascular FAI was associated with noncalcified atherosclerotic plaques and was increased in culprit lesions of patients with ACS [71]. In the Cardiovascular RISk Prediction using Computed Tomography (CRISP-CT) study, the perivascular FAI around the right coronary artery could predict all-cause and cardiac mortality at a cutoff of ≥−70.1 HU [72]. Importantly, this imaging marker had additive, incremental prognostic value on top of established CV risk factors [72]. Compared to the traditional high-risk features mentioned above, (positive remodeling, low-attenuation plaque, spotty calcification or napkin-ring sign), the superiority of perivascular FAI at the previously established cutoff was established [73]. The regression of perivascular FAI may also be used as a guide for patient response to statin therapy [74].

### 4.4. CT Myocardial Perfusion

In the presence of obstructive CAD lesions in plain CCTA, the determination of their hemodynamic severity represents a reasonable next step. Qualitative evaluation through static CT myocardial perfusion (CTP) is based on a snapshot of myocardial iodine distribution at one time point during first-pass perfusion. Low enhancement areas are compared with either remote myocardial segments or are normalized to the left ventricle attenuation. The reversibility of the segmental ischemia is assessed through a comparison of stress and baseline perfusion. Several drawbacks can be identified, including the reliance on the presence of normally perfused segments and the mistiming of contrast bolus due to the effect of cardiac output and contrast flow rate. Moreover, breathing, high heart rate and beam hardening artifacts may be misinterpreted as perfusion defects. At the same time, in the case of balanced ischemia, myocardial hypoperfusion may not be detected. On the other hand, dynamic CTP, based on serial images of myocardial contrast inflow to construct time-attenuation curves, allows for a quantitative evaluation of myocardial perfusion.

At present, however, the role of CTP has not been firmly established. According to a recently reported study, CTP provided incremental information regarding the hemodynamic significance of CAD compared to plain CCTA, with invasively measured FFR being the reference standard [75]. This was especially important in the cases of the 2-vessel and 3-vessel disease. Importantly, this study enrolled subjects with known CAD and even prior percutaneous revascularization. The authors proposed a relative myocardial blood flow (MBF) cutoff of 0.71 for the identification of hemodynamically significant stenosis. Concerning patients with stable angina pectoris, the diagnostic performance of CT-MBF was similar to CMR perfusion and superior to static CTP and plain CTA [76]. In the Dynamic Stress Perfusion CT for Detection of Inducible Myocardial Ischemia (SPECIFIC) study, the use of dynamic stress CTP in patients with suspected CAD was superior to plain CCTA, especially in vessels with moderate stenosis (50–69%) [77]. Regarding its prognostic significance, the mean MBF of all ischemic segments was the most potent predictor of 1-year major CV events in 142 patients with chest pain and intermediate-to-high pre-test probability for CAD [78]. Other parameters that were evaluated were CAC score, CT-FFR, and high-risk plaque features. To sum up, the need for unnecessary invasive procedures may be reduced through a strategy involving CCTA and dynamic CTP [79]. Dynamic CTP performance in cases of multivessel CAD also appears appropriate prior to referral for invasive coronary angiography.

### 4.5. FFR_CT_

The functional significance of moderate stenoses can be non-invasively determined with the use of FFR_CT_. This method uses a patient’s standard cCTA study along with a fluid dynamics model to calculate a value that can be interpreted similarly to an invasive FFR measurement. A FFR_CT_ ≤ 0.8, indicative of ischemia, was associated with a higher total and calcified plaque volume, plaque length, and luminal stenosis >50% derived from CCTA [80]. A plethora of clinical studies has been performed to assess its correlation with the gold-standard invasive coronary angiography-derived FFR, its superiority to CCTA, and to evaluate its prognostic significance. With invasively determined FFR as a reference standard, FFR_CT_ on top of CCTA has superior specificity and positive predictive value compared to CCTA alone in the per-vessel analyses [81]. The AUROCs were significantly higher in both the per-patient and per-vessel analyses. In patients with stable angina and an abnormal single photon emission computed tomography who were referred for invasive coronary angiography, FFR_CT_ was sensitive and specific at both the patient and vessel level, especially in the left circumflex artery (sensitivity: 83%, specificity: 92%) [82]. Importantly, an invasive procedure could have been avoided in 53% of the patients, should the FFR_CT_ have been preferred. The diagnostic accuracy of FFR_CT_ has been tested also in individuals with extensive coronary calcification (CAC score > 399) [83]. Such patient populations frequently have FFR_CT_ ≤ 0.8, while those that had higher values exhibited few major adverse CV events during the short-term follow-up [83]. No impact of coronary calcification severity has been noted on the diagnostic accuracy of FFR_CT_ [84]. A recently reported systematic review and meta-analysis highlighted the prognostic significance of FFR_CT_, since patients with stable CAD and negative FFR_CT_ (>0.8) displayed a low rate of adverse CV events [85].

Machine learning algorithms have also been introduced to enable on-site FFR_CT_ estimation. According to published studies, their accuracy is superior to plain CCTA in identifying hemodynamically significant stenoses [86]. In stable patients with intermediate stenoses, machine learning-based FFR_CT_ could adequately identify hemodynamically significant lesions, as shown by the lower rates of invasive coronary angiography and the higher revascularization-to-angiography ratio compared to CCTA alone [87]. Moreover, it had a greater outcome prediction. Machine learning-based FFR_CT_ could be of value in the preprocedural planning of transcatheter aortic valve replacement as it has the potential to reclassify patients with anatomical signs of obstruction, thus avoiding the need for invasive coronary angiography in a considerable proportion of patients [88,89,90]. Critically, a virtual planner based on FFR_CT_ has been recently investigated, with acceptable accuracy and precision in predicting post-percutaneous revascularization FFR, even in cases of high calcium and disease burden, thus enabling the adequate preprocedural prognostication of patients submitted to percutaneous revascularization [91].

## 5. Magnetic Resonance Imaging (MRI)

### 5.1. General Aspects of Cardiac MRI Approach

Cardiac magnetic resonance imaging (MRI) is produced by the hydrogen nuclei of the tissues. Indeed, the advantage of cardiac MRI is based on the different content of tissues in hydrogen and the difference in the associated paramagnetic properties of adjacent molecules which allow the differentiation of tissues according to their composition [92]. However, the small excess of hydrogen nuclei capable of generating a signal in cardiac MRI decreases the signal-to-noise ratio which is overcome with anisotropic voxels volumes of 1.5 mm × 1.5 mm × 8 mm as far as regarding 1.5 Tesla main magnetic field systems [93].

Anatomic evaluation of the coronary artery tree is the most straightforward and comprehensive for human perception as it is achieved with invasive angiography of the coronary arteries which is considered the ground truth for appraisal of coronary lesions and stenosis. However, with epicardial arteries diameter from 500 μm to 5 mm [94,95] and the anisotropic high-volume voxels in 2-dimensional standard cardiac MRI application, a reliable evaluation of coronary artery tree cannot be achieved.

### 5.2. Free-Breathing Coronary Cardiovascular Magnetic Resonance Imaging

Current electrocardiogram triggered free breathing 3-dimensional applications of cardiac MRI with a 2-dimensional navigator in the right hemidiaphragm—to compensate for the respiratory motion of the heart—and with a 3 Tesla main magnetic field can achieve isotropic voxel volumes of 0.9 mm^3^ [96]. Although the acquired spatial resolution is significantly improved from the standard 2-dimensional cardiac, MRI cannot reach the spatial resolution of invasive coronary angiography ~0.2 mm [97]. However, the most proximal segments of the coronary arteries can be visualized with adequate accuracy, especially regarding sensitivity and negative predictive values and with moderate positive predictive values compared to invasive coronary angiography [96]. However, the positive predictive value is moderate since cardiac MRI may overestimate stenosis and especially in the territory of the left circumflex coronary artery, which is far from the surface coil and there is no compensation for its movement to the anteroposterior plane.

The limitation of the coronary Cardiovascular MRI may be overcome since a parallel CV assessment of the myocardial synthesis, of left ventricular function, of myocardial viability and of myocardial ischemia through functional tests can be achieved. The integration of coronary arteries’ anatomic features with this morpho-functional information may improve diagnostic ability and decision-making. Regarding coronary computed tomography angiography coronary CV MRI presents less artifacts in heavily calcified coronary arteries and does not suffer from the disadvantages and complications of iodinated contrast agents [98].

### 5.3. Atherosclerotic Plaque Characterization with Cardiac MRI

Over the last few years, attention has been shifted to plaque morphologic characteristics since the relationship between CV prognosis or acute coronary syndromes with a degree of coronary stenosis is not straightforward but rather multifactorial [99]. Indeed, the atherosclerotic burden and calcification of coronary arteries, as well as features of atherosclerotic plaques that we used to call “vulnerable plaque”, may provide a better prognosis of events [99,100,101,102]. However, the question remains if cardiac MRI may identify vulnerable atherosclerotic plaque characteristics. At first, with free-breathing cardiac MRI sequences, it has been documented that coronary plaques may be visualized in high resolution [103]. High-intensity plaques based on T1 weight cardiac MRI images and especially when the plaque to myocardium ratio exceeds 1.4 has been found as an independent predictor of coronary events [104]. The intensification of the T1 weight signal is classically considered when there is an excess of water hydrogen in the tissues, especially in cases of edema. In the carotid atherosclerotic plaques, T1 weight images have been found to be intensified in regions of intraplaque hemorrhage [105]. Interestingly, studies have also previously documented that high-intensity T1-weight coronary plaques are associated with positive remodeling of the artery, ultrasound attenuation and low CT density [106]. From a theoretical standpoint, late gadolinium enhancement in a fibrous cap may be identified but in vivo studies do not document any discriminative ability or prognostic value for acute coronary syndromes based on the pattern of atherosclerotic late gadolinium enhancement [107]. Another approach, with the combination of 3-dimensional bright blood and black blood phase-sensitive inversion recovery coronary angiography, can be used to visualize thrombus and intraplaque hemorrhage [108,109].

### 5.4. Myocardial Perfusion Images and Assessment of Myocardial Ischemia with Cardiac MRI

Ischemic heart disease is wide-ranging from metabolic abnormalities of myocardial cells to contractility abnormalities, regional wall motion abnormalities and myocardial necrosis. Cardiac MRI can investigate and reveal different stages or patterns of ischemic heart disease.

**Figure 3:**
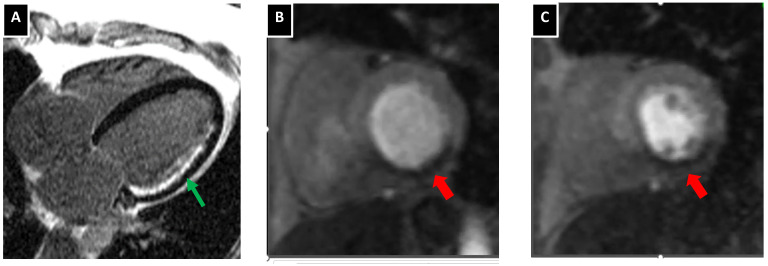
Cardiac magnetic resonances imaging provides information on myocardial viability (panel (**A**)) with the extent of late gadolinium enhancement (red arrow) compared to the total myocardial thickness correlating inversely with viable myocardium. With first-pass perfusion studies following vasodilatation, ischemia can be diagnosed as a region lacking enhancement during early gadolinium studies green wide arrows in the region of basal (panel (**B**)) and mid (panel (**C**)) inferior and inferolateral myocardial wall.

Myocardial contractility can be reliably assessed with cardiac MRI with excellent interobserver and intra-observer variability (Figure 3) [110]. Beyond left ventricular volumes and left ventricular ejection fraction myocardial strain algorithms can be used to further quantify myocardial systolic performance [111].

Monitoring of the first pass of contrast medium through the heart is used to assess myocardial perfusion. In resting conditions, myocardial perfusion defects are recognized when the coronary artery is over 85% stenosed. However, the limited vasodilatory capability of coronary segments with stenosis exceeding 50% can reveal myocardial perfusion defects when a vasodilatory stimulus such as adenosine is applied [112]. Several studies have documented the feasibility of the technique to functionally assessed myocardial ischemia. In a recent metanalysis comparing cardiac imaging methods to fractional flow reserve results, cardiac MRI proved to have the highest performance for diagnosis of coronary artery disease and myocardial ischemia compared to other imaging modalities such as single-photon emission computed tomography and stress echocardiography [113]. Indeed, the sensitivity of MRI was estimated at 90% and the specificity at 94%. Moreover, a positive stress cardiac MRI test has prognostic ability in patients with chest pain and coronary stenosis of unknown significance in cardiac computed tomography angiography [112].

Regarding ischemic heart disease detection of myocardial viability is of importance since may guide treatment decisions. Dobutamine stress cardia MRI when identifying contractile reserve can be used as a surrogate of myocardial contractility however its sensitivity is low [114]. Interestingly late gadolinium cardiac MRI can reliably (compared to glucose uptake positron emission tomography studies) identify segments with a myocardial infarction and are not viable especially when the transmural extent of late gadolinium enhancement is more than 50% [115].

### 5.5. Prognostic Role of Cardiac MRI in Post-Acute Coronary Syndrome Patients

In post-acute coronary syndrome patients, cardiac MRI can provide useful information regarding the extent of myocardial infarction, as assessed with late gadolinium-enhanced imaging, the left ventricular ejection fraction, and the regional wall motion abnormalities. Among other parameters, cardiac MRI, with the use of contrast agents, can recognize myocardial territories with microvascular obstruction associated with the no-reflow or slow reflow phenomenon. Importantly, the extent of microvascular obstruction adversely affects a long-term prognosis in addition to the established risk scores [116].

## 6. Stress Echocardiography

Stress echocardiography was developed as a reliable and affordable tool for the identification and risk stratification of individuals with suspected or established coronary artery disease (CAD).

**Figure 4:**
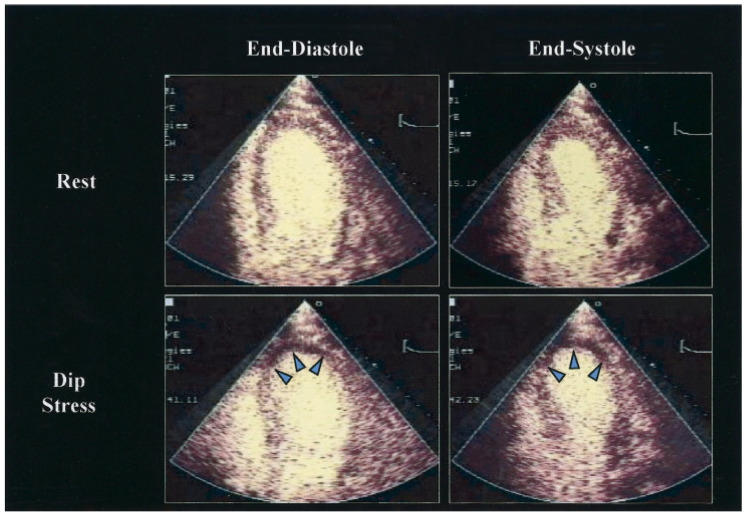
Dipyridamole (Dip) stress contrasts echocardiography using real-time perfusion imaging at a low mechanical index, showing images at end diastole and end systole in a patient with significant stenosis of the left anterior descending coronary artery. Note the perfusion defect that developed in the apex (highlighted by arrows) and the corresponding wall motion abnormality. Reprinted from Journal of the American College of Cardiology, Volume 45, Issue 11, Armstrong, W.F. et al. [122], Stress Echocardiography: Current Methodology and Clinical Applications, pages 1739–1747, Copyright 2005, with permission from Elsevier.

The identification of novel or worsening wall motion anomalies with stress is essential for the diagnosis of myocardial ischemia by stress echocardiography [117]. For the diagnosis of severe CAD, the sensitivity and specificity are 85% and 77%, respectively [118]. Even when the pretest probability is moderate or high, patients with negative stress echocardiography have very low mortality and a number of major events (0.6–0.8% per patient/year) [119]. In contrast, pathological stress echocardiography test predicts outcomes that are significantly poorer than normal stress tests (survival 71.2 vs. 92%, respectively) [120]. In addition, stress echocardiography offers prognostic information beyond clinical data (gender, age, heart failure treatment) or resting left ventricular function [121] (Figure 4).

### 6.1. Additional Modalities to Improve Diagnostic Performance of Stress Echocardiography

#### 6.1.1. Enhanced Stress Echocardiography with Contrast

The intravenous administration of contrast substances during stress echocardiography is frequently utilized. Microbubbles, which are composed of a gas-filled core of either air, nitrogen or an inert gas with high molecular weight, are used as contrast substances. By boosting backscatter in an ultrasound spectrum, microbubbles produce contrast. As a result, myocardial tissue and blood are more easily distinguished from one another, and the endocardial line is more clearly defined.

Due to contrast agent use, stress echocardiography is now possible even in morbidly obese patients [123]. Because the early information on contrast safety was contradictory, extensive investigation studies have been carried out and demonstrated that the use of contrast media is not only clinical standard practice, but also aids in the recognition of significant irregularities that otherwise would have gone unnoticed [124].

In order to identify inducible wall motion anomalies, the sensitivity and specificity of stress echocardiography depend on the ability to clearly visualize the whole left ventricle endocardial borders. Particularly in single-vessel CAD, left ventricular opacification raised the stress echocardiography‘s sensitivity from 80 to 91% [125]. With the introduction of a contrast agent, interpretation with high confidence rises from 36 to 74%. A stronger effect of contrast was noticeable when endocardial vision and confidence in interpretation were diminished in unenhanced pictures [126]. Interobserver agreement rises when contrast is used (79 vs. 69% in images without contrast enhancement). Even for inexperienced operators, contrast-enhanced stress echocardiography increases the agreement between their interpretation and that of the knowledgeable reader [127,128].

In addition, myocardial perfusion imaging has been shown to be more useful than wall motion analysis in predicting patient outcomes in single-center studies using dipyridamole stress, treadmill activity, bicycle stress and dobutamine [129,130]. When there were no abnormalities in the wall motion in any of these circumstances, delayed replenishment of the contrast during a continuous infusion of microbubbles was observed in a considerable percentage of patients and seemed to have independent prognostic value.

#### 6.1.2. Real-Time Three-Dimensional Imaging for Stress Echocardiography

Two-dimensional stress echocardiography has a number of drawbacks, such as a delay in obtaining all pictures while the heart rate is at its highest and foreshortening of the left ventricle [117,131]. The most recent imaging technique, real-time three-dimensional (3D) stress echocardiography, can be used to overcome these limitations. With the advent of matrix array transducers, 3D echocardiographic pictures may be captured in real-time, enabling high-definition imaging of the beating heart [132]. Both a multi-beat recording and, more recently, a single-beat acquisition can be used to obtain real-time full-volume 3D data sets for stress. With more recent transducers and software, a full-volume 3D data set can be acquired in its whole in a single beat without stitching or other artifacts.

The use of real-time 3D stress echocardiography helps deal with some of the drawbacks of conventional 2D stress echocardiography. At first, we can obtain pictures while the heart rate is still at its highest limits [133]. A sudden drop in heart rate during peak stress may result in the acquisition of some views below target heart rates, decreasing the overall sensitivity in identifying myocardial ischemia, since multiple acquisitions from multiple windows are required in 2D to image all myocardial segments. However, a full 3D data set can be collected from an apical window, and images can be edited to look at different planes or slices from the same heartbeat, increasing the sensitivity of identifying irregular wall motion.

Another issue with reading 2D-stress echocardiography is that the myocardium’s 2D slices, when viewed pre- and-post stress, can occasionally be out of alignment. The sensitivity and specificity for detecting myocardial ischemia may be reduced as a result of the mismatch of the myocardial segments and the foreshortening of the LV. However, real-time 3D stress echocardiography enables a visual evaluation of the complete real LV by cropping the volumetric data sets along the appropriate axes [134]. With the help of the 3D volumetric data set, it is possible to compare myocardial segments more analogously and without foreshortening.

Comparing RT-3D-DSE and 2D DSE, there is comparable accuracy between the two modalities but due to improved imaging in the apical segments with the latter, there is improved sensitivity in the left anterior descending coronary artery territory [135].

The diagnostic potential of 3D-stress echocardiography as a tool in the evaluation of suspected CAD will continue to increase due to ongoing technical advancements. The image quality of 3D-stress echocardiography will be enhanced by single-beat capture, lower footprint matrix transducers, broader sector angles, and higher frame rates [136].

#### 6.1.3. D Strain-Speckle Tracking Imaging in Stress Echocardiography

With the recently developed strain/strain rate analysis, myocardial deformation is directly assessed using tissue Doppler imaging, which is not volume dependent and is assessed irrespective of myocardial tethering or translational effects. Throughout the cardiac cycle, the movement of speckles in a particular area of concern is tracked frame by frame. The key benefits of speckle tracking echocardiography are its lack of angle dependency, great deliverability and repeatability. The use of speckle tracking in stress echocardiography is still up for controversy since it lacks standardization and/or reference cutoffs and heavily relies on the operator’s experience [137]. Its viability may also be constrained by high heart rates, low-quality acoustic windows, the relatively low frame rates (50–90 frames/s) and inter-vendor heterogeneity [138]. However, as of now, the evidence for its application in clinical practice is growing [139].

The systolic longitudinal and circumferential shortening and radial thickening, during ischemia are diminished or absent. Even though there is not currently a clear cut-off value, a considerable reduction in regional and global longitudinal strains from rest to stress has been shown to be consistent with myocardial ischemia [138] because longitudinal subendocardial fibers are impacted early in the case of myocardial ischemia. According to a study by Rumbinaitė et al., the global longitudinal strain had an AUC of 0.95 (sensitivity 94%, specificity 92%) for detecting severe coronary artery disease, which was defined as stenosis with a diameter of 70% or more on coronary angiography and was shown to be hemodynamically important by adenosine CMR [140]. In addition, the recovery of the left ventricular global longitudinal strain was the best indicator of obstructive CAD and it was connected to the results of positron emission tomography (the extent, localization, and depth of myocardial ischemia) [141].

The main drawbacks are the global longitudinal reduction during SE, even in healthy people, as a result of modifications in the loading parameters (reduced LV preload, increased systolic pressure), and the insufficient frame rate when tachycardia is present [142].

### 6.2. Coronary Flow Reserve in Stress Echocardiography

The American Society of Echocardiography and the European Association of Echocardiography both recognize the coronary flow reserve (CFR) assessment technology, which has been in use for more than 30 years, as an established non-invasive modality for assessment of both microvascular and macrovascular, functionally significant coronary disease [143,144]. Doppler echocardiography is used to noninvasively assess the CFR, which is possible in the left anterior descending artery (LAD) in >90% of patients, and the posterior descending artery (PDA) and left circumflex artery (LCx) in about 50% of patients [145,146]. The approach is made even more feasible by using contrast agents.

Maximum diastolic blood flow in the coronary artery is measured both at rest and following administration of adenosine (2 mg bolus or 140 mcg/kg/min infusion over 2–3 min), regadenoson (a single slow bolus), dipyridamole (0.84 mg/kg over 6 min) or dobutamine (typically at 30 mcg/kg/min). When dobutamine is used, the test is considered sufficient for CFR analysis if the heart rate increases by 50 bpm from baseline or by at least 75% of the highest anticipated HR [138]. It is recommended that the measurement be conducted across at least three cycles, with the average value used. Maximum diastolic pressure during hyperemia divided by maximum diastolic pressure at rest reflects the CFR. When adenosine is given immediately prior to the test or when dobutamine is used during the test, CFR might be combined with stress echocardiography to obtain additional information. The relevance of CFR in the diagnosis of CAD and its additive predictive value in dobutamine stress echocardiography is well supported by evidence.

In a Substudy of the Randomized Compare-Acute TrialIn Haeck et al., studied the relationship between CFR and clinical outcomes in Fractional Flow Reserve-positive lesions when treated medically, and also the impact of percutaneous coronary intervention vs. medical therapy on the management of FFR-positive lesions with a preserved CFR. They found that clinical results in patients with positive FFR were unaffected by preserved or low pb-CFR. Additionally, when treated medically vs PCI, patients with FFR-positive coronary lesions but a preserved CFR experienced more clinical events [147].

### 6.3. Myocardial Viability Assessment

Nonrandomized retrospective research from the 1990s to the early 2000s demonstrated that revascularization of patients with viability was associated with improved results compared to conventional treatment in those with ischemic left ventricular dysfunction. Stress echocardiography with low-dosage dobutamine is used to induce a contractile response in viable segments with systolic failure. Viable myocardium with a contractile reserve in at least five segments improves the likelihood of functional recovery after coronary revascularization.

In comparison to patients with irreversible contractility dysfunction, patients with significant ischemic left ventricular systolic dysfunction and contractile reserve have reduced perioperative mortality, increased improvements in regional and overall contractile function after the invasive treatment, fewer heart failure symptoms, and in general, lower mortality.

It is noted that in recent studies the ejection fraction and wall motion score are validated options to rely on a bipolar, five-segment definition of viability for the assessment of the global contractile response to dobutamine [148,149,150]. Patients who are candidates for revascularization and have serious global dysfunction, heart failure symptoms, and multivessel disease may still find low-dose dobutamine echocardiography to be helpful [151,152].

Additionally, it has been demonstrated that low-dose dobutamine echocardiography is an effective predictive test in patients who are candidates for cardiac resynchronization therapy. An increase in LVEF or an improvement in wall motion score were used by Kloosterman et al. in a meta-analysis to identify a positive response to the low-dose dobutamine test. This implies that patients who have contractile reserve have viability and that resynchronization enhances overall performance [153].

## 7. Nuclear Imaging

### 7.1. Cardiac Positron Emission Tomography

#### 7.1.1. Vulnerable Plaque Imaging

Although not readily available, nuclear imaging studies have been more frequently used in the latest years in the setting of CVDs. Early studies with 18-fluorodeoxyglucose (FDG) cardiac positron emission tomography (cPET)/CT in cancer patients identified a significant association of the target-to-background ratio (TBR) in the region of the left anterior descending artery with CV risk factors, pericardial fat volume and calcified plaque burden [154]. 18-Fluorodeoxyglucose (18-FDG) cPET highlights the atherosclerotic plaque’s metabolic activity, indicative of inflammation [155]. An important drawback of this method, however, is the myocardial uptake of FDG, which may be counteracted by consuming a low-carbohydrate, high-fat meal the night before the procedure, as also shown by a randomized trial [156,157]. Other approaches, such as the euglycemic-hyperinsulinemic clamp can be applied accordingly, aiming at improved image quality [158].

Other than vascular inflammation, calcifications and microcalcifications could be detected by the use of an alternative radiotracer, 18-sodium fluoride (NaF). Based on a lower myocardial uptake, and with the added motion correction, an enhanced plaque visualization may be achieved, through a 46% reduction in image noise [159]. In clinical studies, NaF uptake was higher in CAD patients and was significantly associated with the CAC score [160]. Moreover, fluoride-positive plaques were more common in ACS patients in comparison to stable CAD [161]. Investigators have also assessed another metric, the coronary microcalcification activity (CMA) across the entire coronary circulation, in patients with recent ACS and multivessel CAD [162]. Even though low-attenuation, vulnerable plaques had elevated CMA and TBR, a CMA of >0 had superior diagnostic accuracy (sensitivity and specificity of 93.1% and 95.7%, respectively) compared to TBR > 1.25 [162]. Novel advances in radiotracer development have been noted, such as (68)Ga-DOTATATE (binds to somatostatin receptor 2 that is expressed in macrophages) [163], (68)Ga-pentixafor (binds to the CXC-motif chemokine receptor 4) [164], 18F-GP1 (binds to glycoprotein IIb/IIIa-receptor) [165] and selective tracers targeting vascular cell adhesion molecule-1 or MMP-13 [166,167]. The combination of cPET with MRI for the evaluation of CAD is under investigation and could be of value in the future. Evidence is scarce to this point and the only clinical study to date utilized 18-NaF in gadobutrol-enhanced cPET/MRI. The authors determined that the segmental TBR values of >1.28 and >1.25 could detect TCFAs and lipid cores, respectively [168].

#### 7.1.2. Myocardial Perfusion Imaging

Stress and rest myocardial perfusion imaging (MPI) can be achieved with cPET, through qualitative evaluation of perfusion defects, as well as quantitative by assessing the myocardial blood flow (MBF) at rest and stress, ultimately calculating the myocardial flow reserve (MFR). A reduced MFR, in particular, is a poor prognostic indicator since it was associated with all-cause mortality [169,170]. Semi-quantitative methods of ischemia assessments also exist, such as the summed difference score (SDS) and the summed stress score (SSS). PET may be especially useful in patients with balanced ischemia (multivessel CAD) or moderate stenosis of undetermined hemodynamic significance. The most frequently used radiotracers are 82Rb and 13N, with 15O[H_2_O] and 18F-flurpiridaz being alternatives. The diagnostic accuracy of PET MPI is exceptional (Sensitivity: 89%, Specificity: 90%) [171], and may be superior to single photon emission computed tomography (SPECT) [172,173]. Importantly, patients exhibit lesser radiation exposure, and the examination time is significantly lower. However, a recently reported randomized clinical trial of patients undergoing MPI studies (PET or SPECT) found no differences in the rate of coronary angiography, coronary revascularization, and health status at a 12-month follow-up [174]. It should be noted that high-risk features of PET MPI were associated with a higher need for invasive management compared to the high-risk features of SPECT. An ischemia threshold of 5% on PET MPI may be indicative of the need for early revascularization, since such patients experienced a survival benefit [175]. Due to the inherent disadvantages of PET MPI, including the high cost of cameras, cyclotrons or generators, and the short half-life of tracers, this method is not largely available in most situations.

### 7.2. Cardiac Single Photon Emission Computed Tomography

**Figure 5:**
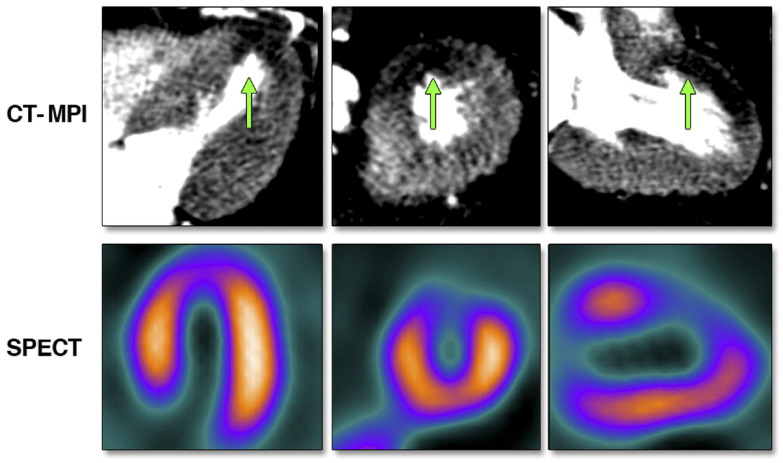
Stress perfusion defect, indicated by the green arrows, in the mid- and distal anterior wall during adenosine stress computed tomography-myocardial perfusion imaging (top) and the perfusion defect in the same wall segments using single photon emission tomography-myocardial perfusion imaging (bottom). Reprinted from JACC: Cardiovascular Imaging, Volume 3, Issue 10, Tamarappoo, B.K. et al. [177], Comparison of the Extent and Severity of Myocardial Perfusion Defects Measured by CT Coronary Angiography and SPECT Myocardial Perfusion Imaging, pages 1010–1019, Copyright 2010, with permission from Elsevier.

Cardiac SPECT is a frequently performed non-invasive imaging procedure in the routine clinical practice of patients with or suspected CAD (Figure 5). Through the flow-dependent or metabolism-dependent uptake of a radiopharmaceutical by the functional myocardium, myocardial perfusion can be assessed at rest and after exercise or pharmacological stress. Gamma radiation is converted to an electrical signal through a recently developed cadmium-zinc-telluride detector, with increased sensitivity to detect obstructive CAD [176]. It can be employed in the setting of suspected or documented CAD with non-acute symptomatology, and for its risk stratification. It can be helpful in evaluating and relatively quantifying perfusion, as well as the simultaneous evaluation of myocardial wall motion. SPECT can provide additional prognostic information on top of clinical evaluation, while it may direct treatment decisions.

Contemporary studies have demonstrated the prognostic significance of cardiac SPECT. SPECT myocardial perfusion imaging provided incremental prognostic information on top of anatomic CCTA evaluation regarding adverse outcomes [178]. In a large cohort of patients undergoing SPEC phase variables (phase entropy, bandwidth and SD) were independently predictive of incident major adverse cardiovascular events and improved the risk stratification provided by perfusion and left ventricular ejection fraction [179]. Another study highlighted that patients with a summed stress score of >8 had the lowest survival [180]. Moreover, revascularization in such patients may confer a survival benefit compared to optimal medical management [180]. At the same time, individuals with a summed stress score of ≥8 had a better prognosis independently of the treatment approach [180]. Concerning specific patient populations, the use of SPECT in individuals with diabetes mellitus may be important in guiding revascularization at a low ischemic threshold (>8.6%) as this can lead to lower mortality rates [181]. Importantly, diabetic patients with a completely normal study are expected to have a very low adverse outcome rate [182]. SPECT may be of value in patients with non-obstructive CAD, since an abnormal study could indicate a similarly poor prognosis to that of patients with obstructive CAD [26].

In conclusion, the introduction of dedicated cameras outfitted with solid-state detectors as the new norm has enhanced cardiac SPECT. This implementation contains well-established, quantitative diagnostic and prognostic parameters of relative regional ischemia, allowing the quick assessment of myocardial perfusion and function with exposure to a modest dose of radiation. The quantification of MBF and CFR will come in the following stage. Due to the new cardiac camera systems’ higher temporal resolution and greater sensitivity, the approaches are easily adapted from PET, which serves as the industry standard, and applied to the area of SPECT.

## 8. Comprehensive Approach

Since most imaging methods address the same question, there is considerable overlap in their use and their value regarding the evaluation of patients with CAD. Accordingly, the choice of one method over another should be based on patient’s specific characteristics, on the clinical question under investigation, on the accuracy and specificity of the method and, of course, on local availability and experience.

At this point, we can distinguish two clinical scenarios. Firstly, the evaluation of a patient with suspected CAD and unknown coronary anatomy. The other one is the evaluation of patients with known coronary anatomy of unknown functional significance.

In the case of patients with suspected CAD, the imaging method should be selected on the base of pre-test probability and the accuracy of the method. Characterization of the symptoms as angina, atypical angina and non-angina is of importance and based on the age and sex and according to circumstances based on other modifiers of pre-test probability, we can separate the patients into low moderate or high pre-test probability [183,184,185].

For simplicity reasons anatomical evaluation, with the most clinically used CCTA, is preferentially applied when there is a low likelihood of CAD [186]. Noninvasive functional tests with high accuracy, specificity, and positive predictive value such as stress echo, SPECT and stress cardiac MRI have a better rule in performance and are used when there is a high clinical likelihood. Another clinical use of the later imaging modalities is the assessment of viability [187,188,189]. However, the most accurate test is difficult to determine since there is a lack of ample head-to-head comparisons. Knuuti J et al., in a large meta-analysis comparing the specificity and sensitivity of several functional and anatomical non-invasive tests, provide a higher positive predictive value or rule-in capability for PET and stress cardiac MRI especially when the severity of CAD was confirmed by invasive functional tests [190].

Moreover, the accuracy of the method is affected according to specific patient characteristics while additional information from each modality especially regarding prognostic information can orient clinicians to the proper imaging modality (Table 1). A poor acoustic window signifies the use of stress cardiac MRI while under significant arrhythmia SPECT or PET may be the method of choice. Significant calcification or previous coronary stenting deteriorates diagnostic ability of cardiac CT.

Additional information provided by each modality may also be taken into consideration. CCTA provides not only anatomical information but also morphological characterization of the plaque, may assess FAI, perivascular inflammation and overall cardiac risk while it can also provide perfusion information and functional information [36,38,83,187,191]. Cardiac MRI provides valuable information on the viability of the myocardium, on other myocardial pathologies and, to some degree, morphological information on the atherosclerotic plaques [104,105,115]. Cardiac PET may provide information on vessel inflammation when 18-FDG is used as well as quantitative information on myocardial flow reserve [155,169,170].

## 9. Conclusions

CAD, although theoretically simple regarding functional evaluation and mechanics, does not present a straightforward pattern regarding CV prognosis, events and treatment decisions. Different imaging modalities have evolved over the years offering the opportunity to evaluate noninvasively functional characteristics and to quantify ischemia, to assess viability, to image coronary artery luminal stenosis and to evaluate plaque characteristics. Moreover, additional pericoronary characteristics can be imaged and evaluated regarding inflammation and adipose tissue providing additional information on patients’ prognosis. Since chronic coronary syndromes can be represented by different clinical scenarios, careful selection and combination of different modalities should be applied to achieve the best decision. Over the last few years, stress echocardiography and SPECT proved their value as the most often used but CT and cardiac MRI have been readily introduced into the clinical practice based on their advanced reproducibility and reliability and the multiplicity of different types of information that can offer.

## Figures and Tables

**Table 1:** The role of imaging modalities in the assessment of Coronary Artery Disease.

Imaging Modality	Anatomical Information	Functional Evaluation	Other
CT	+	+	
CCTA	+ Lumen area stenosis Morphological information on plaque synthesis Characterization of vulnerable plaques	−	Perivascular FAI (surrogate of inflammation) Coronary artery calcium (morphological evaluation)
FFR_CT_	−	+ FFR_CT_ ≤ 0.8 is associated with plaque features and at least moderate luminal stenosis Superior to CCTA for the detection of ischemia FFR_CT_ > 0.8 has a high negative predictive value	
CTP	−	+ Static: qualitative evaluation of MP Dynamic: quantitative evaluation of MP Incremental hemodynamic information compared to plain CCTA, especially in multivessel disease CTP-derived MBF flow may be predictive of adverse outcomes	
cMRI	+ Morphological information on plaque synthesis (technically demanding)	+	
Free-breathing coronary cMRI	+ Lumen area stenosis (moderate resolution lower than CCTA)	−	
Stress cMRI	−	+ Excellent sensitivity and specificity for the detection of ischemia	
LGE cMRI	−	−	Viability assessment
Echocardiography	−	+	
Stress Echocardiography	−	+ Hypokinesis detection (usually with dobutamine) perfusion evaluation (with contrast agent) coronary flow reserve (usually with adenosine)	Viability assessment
cPET	−	**-**	Inflammation (with 18-Fluorodeoxyglucose) Calcifications (with NaF) Other atherosclerotic elements (according to tracer)
cPET-MP	−	Qualitative: visualization of perfusion defects Quantitative: calculation of myocardial flow reserve Especially important in multivessel disease and moderate stenosis of undetermined significance Superior accuracy to SPECT	
Cardiac SPECT		Qualitative and quantitative evaluation of MP	

Cardiac computed tomography angiography (CCTA); Cardiac Magnetic Resonance Imaging (cMRI); cardiac positron emission tomography (cPET); Computed Tomography (CT); CT perfusion (CTP); FAI: fat attenuation index; FFR: fractional flow reserve; LGE: late gadolinium enhancement; myocardial perfusion (MP); single photon emission computed tomography (SPECT), (+): the imaging modality provides this type of information (−): the imaging modality does not provide this type of information.
